# Editorial: High-impact respiratory RNA virus diseases, volume II

**DOI:** 10.3389/fvets.2025.1760165

**Published:** 2026-01-08

**Authors:** Victor Manuel Petrone-Garcia, Marco A. Juárez-Estrada, Benjamín Fuente, Inkar Castellanos-Huerta

**Affiliations:** 1 Departamento de Ciencias Pecuarias, Facultad de Estudios Superiores Cuautitlan, Universidad Nacional Autonoma de Mexico (UNAM), Cuautitlán, Estado de Mexico, Mexico; 2 Departamento de Medicina y Zootecnia de Aves, Facultad de Medicina Veterinaria y Zootecnia, Universidad Nacional Autónoma de México, Ciudad de Mexico, Mexico; 3 Facultad de Medicina Veterinaria y Zootecnia, Centro de Enseñanza Investigación en Extensión en Producción Avícola, Universidad Nacional Autónoma de México, Mexico City, Mexico; 4 Department of Poultry Science, University of Arkansas, Fayetteville, AR, United States

**Keywords:** bovine viral diarrhea virus (BVDV), HPAI H5N1, One Health (OH), porcine reproductive and respiratory syndrome virus (PRRSV), recombinant vaccines

Infectious respiratory diseases pose a significant economic and social risk to the veterinary industry, particularly viral diseases. Taken together, these findings underscore the urgent need to strengthen surveillance, improve diagnostic tools, and provide a coordinated One Health response. Their relevance has recently increased due to the emergence of new viral variants of infectious diseases in species such as swine, cattle, and poultry, as well as in small animals. This increase in the prevalence and incidence of RNA respiratory viruses has driven the development of new strategies for study, diagnosis, control measures, and vaccination, opening new areas of research and the implementation of multidisciplinary approaches.

This editorial covers several highly relevant topics, including the evolution of one of the most critical pathogens in cattle production systems, bovine viral diarrhea virus (BVDV). It addresses the impact of massive NS2 gene deletions on viral persistence. Recent analysis of 21 field isolates of the BVDV-2 genotype reveals that this virus generates a considerable number of viral genomes with deletions (DelVGs), especially in the NS2 region, where more than 90% of the deletions are recorded. The most notable finding is that BVDV2c strains can generate DelVGs up to 150 times higher than those of BVDV2a strains. Furthermore, cytopathic strains produce twice as many DelVGs as non-cytopathic strains. These NS2 gene deletions could play a fundamental role in viral persistence, a phenomenon essential for generating persistently infected (PI) animals. These results expand our understanding of BVDV's genetic plasticity and immune evasion mechanisms, demonstrating that the virus creates diversity within the host before establishing a persistent infection (Holthausen et al.). A key issue in the study of viral diseases is the development of more accurate and efficient diagnostic methods, particularly for bovine respiratory syncytial virus (BRSV), another critical area of animal production. Multiplex RT-qPCR for BRSV and BPIV3 is implemented as a diagnostic tool in this case. This assay presents an LOD95 of 164 copies for BRSV and 359 for BPIV3, with high efficiency and specificity. It can detect approximately 2.4 times more BRSV than viral isolation. This offers an ideal solution for high-demand diagnostic laboratories (Zulauf and Pastey).

Regarding viruses affecting the swine industry, one highly relevant respiratory RNA virus stands out: porcine reproductive and respiratory syndrome virus (PRRSV). PRRSV continues to evolve at an alarming rate, with the discovery of a new recombinant HP-PRRSV strain in Jiangxi, China (NC2023). This strain exhibits characteristics such as belonging to lineage 8 of PRRSV-2 and a genome of 15,321 nucleotides, with recombinant regions derived from the JXA1, JXA1-R, and HUN4 strains. This study, particularly regarding the genome, reports 15 unique amino acid mutations and a 395-amino-acid frameshift in the Nsp2 protein, suggesting increased evolutionary complexity of PRRSV and evidence of the simultaneous circulation of multiple viral lineages capable of efficiently exchanging genetic segments (Wang et al.). This underscores the importance of studying this viral type due to its extreme evolutionary capacity and its potential future impact on the swine industry. One of the most relevant topics today is the use of biotechnology to develop recombinant vaccines, which enable vaccination under safe protocols and an efficient immune response. However, as a study presented in this edition shows, the risk of using recombinant vaccines, such as the MLV (L5A) vaccine virus for PRRS, is being redefined. It's recombination between field viruses generated highly virulent strains, such as the GX2024 isolate, which exhibits severe disease and mortality rates of up to 100% in 4-week-old piglets within 14 days (Gao et al.). Recombination between wild-type strains and modified live vaccines to generate extremely pathogenic viruses represents a critical biosecurity issue on pig farms. Among the possible strategies for controlling PRRSV, the use of zinc sulfate in an *in vitro* model (Yang et al.) is being investigated. This demonstrates a reduction in viral replication in the Marc-145 cell model, attributed to the reduction of reactive oxygen species (ROS) and malondialdehyde (MDA), the increase in superoxide dismutase (SOD) and catalase (CAT) activities, and the modulation of the pro-inflammatory cytokines IL-6, IL-8, and TNF-α, with a concomitant increase in the anti-inflammatory cytokine IL-10, as well as a reduction in the activation of apoptosis-related proteins. Therefore, these results suggest a potential therapeutic role for zinc sulfate as an immune modulator and antioxidant during PRRSV infection. Finally, epidemiological analysis is explored, demonstrating its relevance to these viral types through fluid tests, primarily saliva samples from piglet mortality. These tests support the use of tongue fluids from stillborn piglets as a rapid and low-cost surveillance tool (Machado et al.).

On the other hand, another highly relevant respiratory RNA virus mentioned in this edition is avian influenza. The emerging threat posed by HPAI H5N1 clade 2.3.4.4b and its transmission to pigs is explored, as well as the recent detection of the B3.13, D1.1, and D1.2 genotypes of the virus in dairy cows, poultry, wild birds, wild mammals, humans, and, recently, pigs, indicating a significant shift in the virus's ecology. Pigs, recognized as “mixing vessels” for influenza, constitute a potential bridge to the human population. However, knowledge gaps persist, including the lack of systematic studies in pigs, uncertainty about their actual susceptibility, the still-unexplored impact on production, and the risk of recombination with endemic swine influenza. The review emphasizes the urgent need to implement active surveillance and integrated control strategies under the One Health framework (Mena Vasquez et al.). In the same context, other authors analyze a new canine influenza H3N2 virus isolate from China, which revealed amino acid substitutions similar to those observed in human viruses. This finding indicates a risk of interspecies transmission and the potential for the virus to adapt progressively to humans. Therefore, continuous surveillance of companion animals is required Genetic (Li et al.).

The study of alternative diagnostic methods has also provided insights to avoid potential errors or failures in these promising new diagnostic tools, such as anti-dsRNA antibodies, which have limited potential as universal viral detection tools. Despite initial promises of universality and independence from the viral genome, studies have revealed significant limitations in the application of specific diagnostic tools (de le Roi et al.). Inconsistent detection, incomplete localization of viral antigens, and detection even in uninfected tissues have been observed, compromising their reliability as a universal diagnostic tool. Consequently, exploring new markers based on interferon cascades is suggested as a more robust alternative. In environmental virology, a study of 600 pregnant women found a correlation between ozone exposure during pregnancy and a 40% reduction in the risk of c infection in the third trimester. Additionally, the quartile with the highest exposure showed a 99% lower probability of disease (Zhang et al.). While these results are promising, the study authors emphasize caution due to ozone toxicity and the uncertainty surrounding the mechanisms underlying this association.

The review of the presented studies reveals a complex landscape in virology. BVDV demonstrates an extraordinary capacity to generate defective genomes, which contributes to its persistence and immune evasion. PRRSV continues to recombine and evolve into more pathogenic forms, posing a constant challenge to disease control. The proximity of H5N1 to pigs reinforces concerns about the possibility of an avian pandemic, given the virus's high pathogenicity and its potential for interspecies transmission. In this context, emerging diagnostic and therapeutic tools offer a glimmer of hope. The development of markers based on interferon cascades, as mentioned previously, represents a significant advance in the early diagnosis of viral infections. Likewise, research into new antiviral therapies and vaccines is essential to mitigate the impact of emerging viral diseases. Environmental factors, such as ozone exposure, can also unexpectedly influence viral susceptibility, underscoring the importance of a holistic approach to viral disease research and control. This approach, known as One Health, integrates human, animal, and environmental health to address global health challenges more effectively.

In conclusion, the findings presented in this review call for action on several fronts. It is imperative to strengthen biosecurity measures to prevent the spread of viral diseases, increase molecular surveillance to detect and characterize new viral strains, and invest in the development of effective vaccines and antiviral therapies. Furthermore, adopting a One Health approach is crucial to comprehensively address global health challenges and prepare production systems for constantly evolving viral threats.

